# Ferroptosis-based advanced therapies as treatment approaches for metabolic and cardiovascular diseases

**DOI:** 10.1038/s41418-024-01350-1

**Published:** 2024-07-27

**Authors:** Francesca Maremonti, Wulf Tonnus, Shubhangi Gavali, Stefan Bornstein, Ajay Shah, Mauro Giacca, Andreas Linkermann

**Affiliations:** 1 Division of Nephrology, Medical Clinic III, University Hospital Dresden, Technische Universität Dresden, Dresden, Germany; 2 Department of Medicine V, University Medical Centre Mannheim, University of Heidelberg, Mannheim, Germany; 3 Department of Internal Medicine 3, University Hospital Carl Gustav Carus at the Technische Universität Dresden, Dresden, Germany; 4 Diabetes and Nutritional Sciences, King’s College London, London, UK; 5 Center for Regenerative Therapies, Technische Universität Dresden, Dresden, Germany; 6 Paul Langerhans Institute Dresden of Helmholtz Centre Munich at University Clinic Carl Gustav Carus of TU Dresden Faculty of Medicine, Dresden, Germany; 7 King’s College London British Heart Foundation Centre, School of Cardiovascular & Metabolic Medicine and Sciences, London, UK; 8 Division of Nephrology, Department of Medicine, Albert Einstein College of Medicine, Bronx, NY USA

**Keywords:** Translational research, Cardiovascular diseases, Metabolic disorders

## Abstract

Ferroptosis has attracted attention throughout the last decade because of its tremendous clinical importance. Here, we review the rapidly growing body of literature on how inhibition of ferroptosis may be harnessed for the treatment of common diseases, and we focus on metabolic and cardiovascular unmet medical needs. We introduce four classes of preclinically established ferroptosis inhibitors (ferrostatins) such as iron chelators, radical trapping agents that function in the cytoplasmic compartment, lipophilic radical trapping antioxidants and ninjurin-1 (NINJ1) specific monoclonal antibodies. In contrast to ferroptosis inducers that cause serious untoward effects such as acute kidney tubular necrosis, the side effect profile of ferrostatins appears to be limited. We also consider ferroptosis as a potential side effect itself when several advanced therapies harnessing small-interfering RNA (siRNA)-based treatment approaches are tested. Importantly, clinical trial design is impeded by the lack of an appropriate biomarker for ferroptosis detection in serum samples or tissue biopsies. However, we discuss favorable clinical scenarios suited for the design of anti-ferroptosis clinical trials to test such first-in-class compounds. We conclude that targeting ferroptosis exhibits outstanding treatment options for metabolic and cardiovascular diseases, but we have only begun to translate this knowledge into clinically relevant applications.

## Facts

- Ferroptosis contributes to metabolic and cardiovascular diseases and their complications.
- Ferroptosis inhibition represents a promising therapeutic option for several acute diseases.
- Several classes of ferroptosis inhibitors (ferrostatins) are available.
- No anti-ferroptotic therapy has been tested in clinical trials.

## Open questions

- How does ferroptosis contribute to metabolic and cardiovascular diseases?
- What is a reliable biomarker for ferroptosis?
- How can the promising preclinical studies be translated into effective clinical therapies?
- What is the physiological relevance of ferroptosis, and how can potential side effects of ferrostatins be predicted?

## Introduction

Cell death exhibits a hallmark of many diseases. Clinically relevant regulated cell death encompasses apoptosis [1], necroptosis [2], pyroptosis [3] and as an entirely different entity, iron-catalyzed necrosis [4, 5], referred to as ferroptosis [6]. All of these pathways result in a cataclysmic burst [7] mediated at least partially by oligomerization of the plasma membrane protein ninjurin-1 (NINJ1) [8–10]. This ultimate rupture of the plasma membrane defines “necrosis” and is inevitably associated with the release of intracellular content referred to as damage associated-molecular patterns (DAMPs) [11–13] which result in the activation of immune cells in an event defined as necroinflammation [14, 15]. It is beyond the scope of this review to discuss the details of these terms apart from the definition of ferroptosis, and the interested reader is referred to the above cited review articles. However, the potential applications for inhibitors of ferroptosis (ferrostatins) are of particular importance in metabolic and cardiovascular diseases and their complications. Endocrine disorders, with diabetes mellitus as the most prominent example, are particularly susceptible to ferroptosis, and steroid hormones [16, 17] as well as cholesterol metabolites [18, 19] are emerging as important regulators of ferroptosis [20]. Cardiovascular complications, such as myocardial infarction, acute kidney injury and stroke are particularly common in diabetic patients [21], and the associated ischemia-reperfusion injury (IRI) has been a prototype disease model for ferroptosis [5, 22–24]. With lipid peroxidation representing a typical feature of ferroptosis, it is not surprising that fatty liver diseases and IRI in the liver are driven by ferroptosis as well [25, 26]. Please note that while intercellular ferroptosis propagation has been described [27, 28], it remains entirely unclear how cell death propagation between cells is regulated. It is unclear until today to which extent cell death propagation is a specific feature of ferroptosis. We will highlight the potential of advanced therapies targeting ferroptosis and start by defining ferroptosis as iron-catalyzed necrosis.

## Part 1—the definition of ferroptosis

**Fig. 1:**
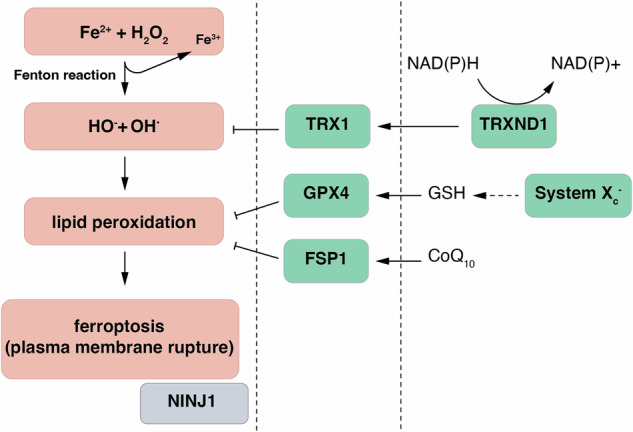
The definition of ferroptosis. By definition, ferroptosis requires an iron-catalyzed reaction with oxygen (Fenton reaction) which is the origin of the ferroptosis reaction cascade. In an intermediate step, reactive oxygen radicals, such as H_2_O_2_ and higher order radicals form as direct and indirect consequences of Fenton reactions. In most cells, thioredoxin (TRX) scavenges such radicals and is immediately regenerated by the potent selenoprotein thioredoxin reductase (TRXRD1) in an NAD(P)H consuming reaction. If such reactive oxygen radicals cannot be controlled, lipids in the plasma membrane (and other intracellular membranes) become peroxidized (Lipid peroxidation). Peroxyl versions of plasma membrane lipids are converted to alcohols by the classical ferroptosis surveillance systems, such as glutathione peroxidase 4 (GPX4) and ferroptosis suppressor protein 1 (FSP1) and others. Lipophilic radical trapping agents compete with lipids for peroxidation, thereby shifting the balance toward lower concentrations of lipid peroxides. Through entirely unknown mechanisms, and potentially involving several undefined intermediate steps, plasma membrane lipid peroxidation results in the oligomerization of pore forming ninjurin-1 (NINJ1) molecules that are required for the cataclysmic burst of the plasma membrane. The rupture of the plasma membrane defines ferroptosis as a necrotic event.

For the purpose of this review article, we define ferroptosis as iron-catalyzed plasma membrane rupture. Fenton reactions lead to the generation of reactive oxygen species that may be controlled by cytosolic redox systems such as the thioredoxin reductase reaction. The thioredoxin-mediated redox signaling represents an antient NAD(P)H-dependent biological reaction pattern, sometimes referred to as the redox metabolome [29], and has been described to be involved in plant immunity [30]. Clearly, the thioredoxin (TRX)-system is involved in the pathophysiology of diabetes as well [31, 32]. Failure of such NAD(P)H-dependent systems to mitigate the cytosolic ROS-concentration triggers the lipid peroxidation and the typical chemical reactions of ferroptosis [33] that are opposed by ferroptosis surveillance enzymes. The best studied system is the glutathione peroxidase 4, a GSH-metabolizing selenocysteine essential for vertebrate life [34, 35]. GPX4 requires direct contact with the plasma membrane to fully function, and mutations in the lipid bilayer anchoring loop of GPX4 result in remarkable dysfunction. Besides GPX4, GSH-independent enzymes have been described to be capable of replacing GPX4 function, at least at the cellular level. Ferroptosis-suppressor protein 1 (FSP1) relies on CoQ10 to prevent lipid peroxidation [36, 37], while other systems, such as membrane-bound O-acyltransferase domain-containing 1 and 2 (MBOAT1/2) are less well understood [38]. With all the chemistry and the ferroptosis surveillance systems studied in detail, the events that connect lipid peroxidation with subsequent rupture of the plasma membrane (necrosis) are almost entirely elusive. Although not all forms of ferroptosis (e.g., RSL3-induced cell death) appear to require ninjurin-1 (NINJ1) [39], recent data have suggested a critical involvement of this molecule to execute the cataclysmic burst of the plasma membrane in ferroptosis through NINJ1 oligomerization [10]. The details of the regulated mechanisms of NINJ1 membrane organization and oligomerization are lacking any concept until today, although it has recently been demonstrated to involve the cutting and releasing of membrane discs [40]. However, inhibition of NINJ1 oligomerization using a monoclonal antibody has been recently demonstrated to prevent other forms of regulated necrosis, such as necroptosis and pyroptosis from their ultimate execution and at least parts of their immunogenicity [9]. Figure 1 demonstrates our current understanding of the ferroptosis-defining cellular reactions. Based on the definition of ferroptosis introduced in Fig. 1, ferroptosis can be interfered with at various levels. Similarly, all conditions that shift the balance toward a higher ratio of lipid peroxidation to ferroptosis surveillance capacity will decrease the threshold for ferroptosis.

## Part 2—approaches to interfere with ferroptosis

**Fig. 2:**
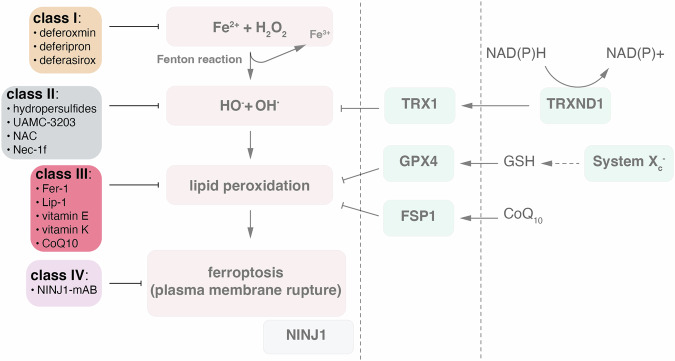
The classes of ferroptosis inhibitors (ferrostatins). Four stages have been defined that characterize the ferroptotic reaction cascade, all of which potentially can be interfered with. In the initial step, Fenton reactions can be targeted by iron chelators, provided that specific cellular compartments, such as the lysosome or the ER, can be accessed by the compound. The default example of this class is deferoxamine (DFO). As soon as free radicals have formed, radical trapping agents (RTAs) may interfere with these short-lived highly reactive products as long as they are available in direct proximity to the radicals. Lipid peroxidation is competed with by lipophilic radical trapping antioxidants (lipophilic RTAs). Prominent examples of this most commonly studied class of ferrostatins are ferrostatin-1 (Fer-1), liproxstatin-1 (Lip-1), 16-86 etc. The ultimate step of plasma membrane rupture can be interfered with by monoclonal antibodies against ninjurin-1 (NINJ1). This step is not specific for ferroptosis, but was initially demonstrated to be required for necroptosis, pyroptosis and even secondary necrosis following apoptosis.

According to the definition introduced in Fig. 1, ferroptosis may be classified into four subcellular stages. We have chosen to allocate inhibitors of ferroptosis (ferrostatins) into four according classes. Ferroptosis inhibitors (ferrostatins) may be subclassified in at least four classes according to their mechanisms of action. Figure 2 demonstrates how these classes relate to the ferroptotic cellular chemical reaction patterns and Table 1 lists several but not all prominent examples according to their mechanism of action. As one example of an innovative approach, the use of selenium-containing Tat-proteins have claimed to protect (e.g., in a stroke model [41]), but the transition of this selenium containing Tat SelPep to other ferroptosis models remains to be demonstrated [41], so we did not add this approach as an individual class of ferroptosis inhibitors here. Other advanced therapies aim at harnessing small non-coding RNAs [42], particularly for cardiovascular diseases, but RNA interference may even sensitize to ferroptosis [43].

**Table 1:** Prominent but incomplete members of the four classes of ferroptosis inhibitors.

	Examples	Mechanism
Class I	- Deferoxamine [45, 63] - Deferiprone [126] - Deferasirox [127]	Iron chelation to inhibit generation of hydroxyl radicals
Class II	- Hydropersulfides [50] - UAMC-3203 [53] - Nec-1f [28] - Omeprazole [54] - Rifampicin [54] - Promethazine [54] - Carvedilol [54] - Propranolol [54] - 7-dehydrocholesterol (7-DHC) [18, 19] - indole-3-pyruvate (I3P) [51]	Detoxify hydroxyl radicals and prevent protein sulfur peroxidation
Class III	- Fer-1 [45] - Lip-1 [34] - Vitamin E [56] - Vitamin K [57] - CoQ10 [36, 37]	Interrupt propagation of lipid peroxidation by detoxifying lipid peroxyl radicals
Class IV	- Monoclonal NINJ1-antibody [9, 10] - Glycine [61, 64, 128]	Prevent cataclysmic burst of plasma membrane

### Class 1 ferrostatins: iron chelators

The requirement of iron is part of the definition of ferroptosis. Erastin was already demonstrated in 2003 to induce rapid cell death in cancer cells in cell culture experiments [44]. Iron chelation using 100 µM deferoxamine (DFO) was demonstrated to protect HT1080 cells from erastin-induced cell death in the first figure of the first ever publication ferroptosis [45]. In the same set of experiments, addition of exogenous free iron potentiated erastin-induced cell death while addition of other divalent metals such as Cu^2+^, Mn^2+^, Ni^2+^, and Co^2+^ did not [45]. However, long before the term ferroptosis was coined, a body of literature on the role of iron and iron chelation in cell death had accumulated as recently reviewed [46]. Importantly, the Fenton chemistry might occur in highly compartmentalized areas of the cell, and iron chelators, chemically, might not reach such areas (e.g., the complex I and III in mitochondria), and therefore might fail to inhibit ferroptosis even though it is mediated by Fenton reactions. While some literature exists that demonstrates iron chelation to protect in disease models, e.g., of traumatic brain injury [47], clinical trials using iron chelators failed to provide clear protective effects e.g., in acute kidney injury [48, 49], potentially because of the specific pharmacodynamics of DFO and derivatives. It will be challenging to design tissue-penetrant iron chelators to target the very upstream reaction of ferroptosis without significant side effect.

### Class 2 ferrostatins: radical trapping agents that function in the cytosolic compartment

Radicals as a result of Fenton reactions may be scavenged by non-lipophilic radical trapping antioxidants. One endogenous example of this class may be hydropersulfide [50]. Additionally, I3P is generated by the secreted amino acid oxidase interleukin-4-induced-1 (IL4i1) the activity of which therefore creates an anti-ferroptosis environment [51, 52]. Pharmacologically, the compound UAMC-3203 [53] was demonstrated to have a favorable PK profile because it is less lipophilic, and therefore appears to function as a particularly potent ferrostatin. Along similar lines, the tissue PK profile of the dual inhibitor of necroptosis and ferroptosis Nec-1f suggests hydrophilic properties while functioning as a low-potency ferrostatin [28]. Several other compounds that have entered clinical routine, such as omeprazole, rifampicin, promethazine, carvedilol and propranolol have been demonstrated to function as ferroptosis inhibitors, while all of these drugs exhibit favorable tissue distribution and therefore might be repurposed as ferrostatins [54]. In this context, it is worth mentioning that rifampicin resistance is a paramount problem in the treatment of mycobacteria [55] (see below).

### Class 3 ferrostatins

#### Lipophilic radical trapping antioxidants

Lipophilic radical trapping antioxidants are by far the best studied class of ferrostatins, and the expanding list of lipophilic RTAs is too long to be listed here. However, ferrostatin-1 (Fer-1) is the first-in-class compound but was ascribed an unfavorable half-life and poor tissue PK [45]. Despite these properties, this small molecule, against expectations, protected mice in several preclinical models including the kidney IRI model which may prolong the half-life of Fer-1 by reduction of its renal excretion [27]. Liproxstatin-1 (Lip-1) protected GPX4-deficient mice from death by acute renal tubular necrosis and therefore is considered a particularly suitable ferrostatin for in vivo research [34]. Vitamin E [56] and Vitamin K [57] are the best studied endogenous representatives of this class of compounds, and at least for Vitamin K, protection from ischemia-reperfusion injury has been reported [57]. Therapeutic supplementation of Vitamin E was tested in patients with nonalcoholic steatohepatitis and was associated with significant improvement compared to placebo or pioglitazone, which was also tested in that trial [58]. While a role for ferroptosis may be questioned in nonalcoholic steatohepatitis, this trial is valuable as it carefully assessed the potential side effect profile of a 96-week episode of ferroptosis inhibition in humans without major untoward effects reported [58]. Vitamin K2 (menaquinone-7) was tested at a dose of 360 µg/day to improve the serum calcification propensity and arterial stiffness in kidney transplant recipients in a single center, randomized, double-blind trial, a 12-week supplementation period, all three severe adverse events were reported to have occurred unrelated to the study medication [59]. Metabolites, such as 7-dehydrocholesterol (7-DHC) [18, 19] can also function as radical trapping agents that oppose ferroptosis. In the case of 7-DHC, genetic deletion of 7-DHC reductase (7-DHCR) was found to protect in two independent ferroptosis screening approaches [18, 19]. While 7-DHCR itself does not function as an RTA, the substrate does. Therefore, the activity level of 7-DHCR indirectly decreases ferroptosis sensitivity, and 7-DHC could be employed therapeutically. The present data on 7-DHC additionally suggest that it could inhibit phospholipid peroxidation both in the FENIX assay (DTUN-induced lipid peroxidation) and iron/ascorbate-induced lipid peroxidation [18, 19]. Therefore, 7-DHC suppresses ferroptosis by diverting the “propagation” of peroxyl radical-mediated damage from phospholipid components to its sterol core, rather than preventing the “initiation” of lipid peroxidation.

### Class 4 ferrostatins: inhibitors of the cataclysmic burst of the plasma membrane

Until today, only NINJ1-oligomerization inhibitors can be allocated to this class [10]. However, it remains to be determined if this approach would inhibit ferroptotic cell death alongside with necroptosis and pyroptosis, but given the overlapping final steps of these pathways before plasma membrane rupture [60], we consider it likely that NINJ1-interference functions as ferroptosis inhibition. Apart from NINJ1, it is known that glycine protects isolated kidney tubules from LDH release [61, 62], and that this effect at least partially contains a ferroptotic component [63]. At least partially, glycine suppresses necrotic cell death by inhibition of NINJ1 membrane clustering [64]. Since glycine does not affect lipid peroxidation directly, it might function in a NINJ1-related way, potentially preventing plasma membrane discs from being shed off the membrane, relating to a recently published concept [40]. Finally, glycine protects kidney tubules from plasma membrane rupture, but the energetic function of these tubular cells is severely compromised, indicating that the cells may be “metabolically dead” while the membrane remains intact [62].

### Conditions that sensitize to ferroptosis

It is beyond the scope of this review to mention the long list of ferroptosis inducers (FINs) that are developed mainly with the intention to drive cancers into ferroptosis. Some commonly used drugs, however, sensitize to ferroptosis by affecting the endogenous surveillance systems of ferroptosis (compare Fig. 1). As an example, dipeptidase-1 (DPEP-1) activity decreases the intracellular GSH pool, thereby decreasing the activity of GPX4. Commonly used steroids, such as dexamethasone and cortisol, through the glucocorticoid receptor, increase DPEP-1 expression and thereby sensitize to ferroptosis and deteriorate ischemia-reperfusion injury [17, 65]. Another recently discovered mechanism of sensitization to ferroptosis involves the emerging treatment with siRNAs. While the approach offers great opportunities to directly target specific proteins by in vivo post-transcriptional gene silencing [66], siRNAs, just like viral RNAs, can be sensed by the mitochondrial protein MAVS [67] and functionally sensitize to ferroptosis independent of the knockdown of the target protein [43]. Most likely by yet another independent mechanism, drugs that induce cell cycle arrest sensitize to ferroptosis and therefore my contribute to its success in tumor therapy [68]. With a perspective to cardiovascular diseases, an oxygen enriched environment, such as it occurs during the postnatal phase, itself induces a cell cycle arrest [69] and thereby may contribute to sensitizing cardiomyocytes to ferroptosis. Finally, iron-selective prodrugs can activate ferroptosis [70], and iron addition of cancers and persister cells in particular [71], can be interfered with in many pharmacological ways and defines a therapeutic approach [72].

## Part 3—metabolic and cardiovascular diseases driven by ferroptosis

The oxygen burst that occurs at the birth of vertebrates creates an environment of hyperoxia in cardiomyocytes which results in cell cycle arrest [69]. It is currently unclear to which extent this potentially priming metabolic event contributes to the outstanding sensitivity of the heart to ferroptosis [73] in diseases such as myocardial infarction [74] and cardiomyopathy [75, 76]. However, the cardiovascular system and its complications emerged as a prime target for treatments with ferrostatins. This also involves the common complications of atherosclerosis, many of which share the common pathophysiological principle of ischemia-reperfusion injury (IRI). Along these lines, kidney IRI has become a classical in vivo setting to study ferroptosis inhibitors [22, 77–79], but liver IRI [34, 57, 80–83], stroke models [84–87] and myocardial infarction and heart failure models [23, 88–96] have been demonstrated to involve ferroptosis and can be improved by ferroptosis inhibition. Consequences of cardiovascular disease-induced necrosis may affect the cardiovascular system itself, as exemplified by cardiac arrythmias. While indeed one study indicated that ferroptosis inhibition may reduce the frequency of atrial fibrillation [97], this topic needs to be studied in much more detail. Most of the existing literature in this field that we will review in the following paragraphs, however, focused on atherosclerosis and patients at risk for cardiovascular complications, such as individuals suffering from diabetes mellitus and/or chronic hemodialysis treatment.

**Fig. 3:**
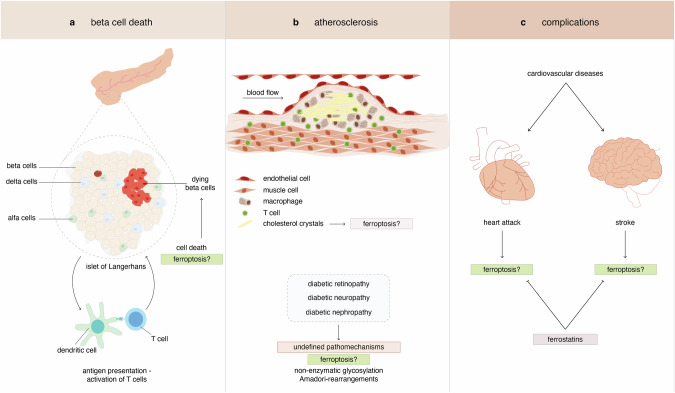
The role of ferroptosis in the pathophysiology of diabetes mellitus. **a** Insulin-producing pancreatic beta cells are highly sensitive to ferroptosis, and their loss is considered the origin of type 1 diabetes mellitus (T1DM), a classical example of a metabolic disease. Diabetes mellitus, not restricted to T1DM, is frequently associated with cardiovascular complications many of which originate from progressive atherosclerotic plaque formation. **b** Cholesterol crystals and cells of both the innate and the adaptive immune systems are involved in atherosclerotic plaque formation, and ferroptosis may be amongst the many pathways that contribute to necrotic debris formation in these plaques. **c** Upon atherosclerotic plaque rupture, commonly observed in patients suffering from metabolic syndrome which includes diabetes mellitus, myocardial infarction, stroke and other disorders associated with a perfusion-deficit or ischemia-reperfusion injury may occur. The necrosis observed in such tissues, particularly its cell death propagation, is known to be driven by ferroptosis. Treatment with various classes of ferrostatins was demonstrated to protect end organ damage in respective experimental models.

The major risk factor for cardiovascular complications, besides cigarette smoking and uncontrolled elevated blood pressure, is diabetes mellitus. As illustrated in Fig. 3, the pathophysiology of disease progression during type 1 diabetes mellitus (T1DM) involves ferroptosis at several different stages. First, pancreatic beta cells, like other hormone producing cells [98, 99], are known to be extraordinarily sensitive ferroptosis, potentially further driven by viral infections [100–103]. Second, atherosclerosis as a major hallmark of diabetic organ complications, involves a necrotic plaque formation the origin of which may comprise of ferroptotic cell death [104–107], potentially driven by cholesterol crystals. Finally, all mentioned ferroptosis-driven IRI complications (Fig. 3c) apply to the classical cardiovascular end points of diabetic patients. T1DM patients are commonly subjected to combined pancreas-kidney transplantation during the process of which ferroptosis and IRI can occur again (see below). Even though the specific literature on ferroptosis in cardiovascular diseases in the setting of diabetes mellitus is limited, some evidence suggests that endoplasmatic reticulum stress and associated ferroptosis are particularly important in myocardial IRI [108].

Prospective cohort studies have investigated cardiovascular diseases and iron uptake in the patient´s diets since the HPFS [109] and NHANES-I [110] studies in 1994. The results of these observational trials have indicated that individuals with relatively high heme iron intake exhibit an increased cardiovascular risk, most prominently represented by increased hazard ratios for coronary heart disease [111]. As it is beyond the scope of this article, the interested reader may be referred to recent reviews on iron uptake in cardiovascular disease [112]. We argue that given the potential deterioration of ferroptosis and additional preclinical experimental data on iron toxicity [53], additional iron supplementation should be used with caution in patients with high risk of cardiovascular complications. This appears to be particularly important for chronic dialysis patients who have lost every trace of renal function, including the production of erythropoietin as the cause of renal anemia. Guidelines for these patients still recommend iron supplementation [113], even though it is well known that cardiovascular complications in dialysis patients are the leading cause of death [114–116]. Given the importance of cardiovascular complications for this patient cohort, we suggest that intravenous iron supplementation must be tested in longitudinal prospective randomized controlled clinical trials designed for cardiovascular complications as the primary end point. In our opinion, without such clinical trials, supplementation of iron to supranormal levels cannot be justified in dialysis patients as long as translational scientific data on ferroptosis are taken into consideration.

Finally, a condition associated with cardiovascular diseases is chronic kidney disease (CKD). It is beyond the scope of this review to list details of CKD pathophysiology. However, most kidney researchers and nephrologists have accepted the general model of acute kidney injury (AKI)-to-CKD transition, the central hypothesis of which interprets CKD progression as repeatedly occurring episodes of AKI which lead to acute tubular necrosis and nephron loss [117]. While it is clear that AKI is mediated by ferroptosis in many scenarios, CKD is commonly associated with Vitamin K deficiency [118] which might further sensitize CKD patients to additional episodes of AKI, thereby driving a vicious circle. Outside the ferroptosis research field, exhaustion of Vitamin K is mostly discussed as a shortage of Vitamin K dependent protein (CKDP) expression that contributes to vascular calcification in CKD patients. These findings are based on the “Rotterdam Study” that demonstrated high menaquinone intake in the diet to be associated with reduced risk of coronary heart disease [119, 120]. In conclusion, all these studies point to a superiority upon ferroptosis inhibition in cardiovascular diseases, and clinical trials with a clearer focus on ferroptosis rather than general vascular outcomes are indicated to assess the clinical applicability of anti-ferroptosis agents. In the following section, we will discuss potential clinical trials to address this question.

## Part 4—considerations on potential clinical trial designs for first-in-class ferroptosis inhibitors

**Fig. 4:**
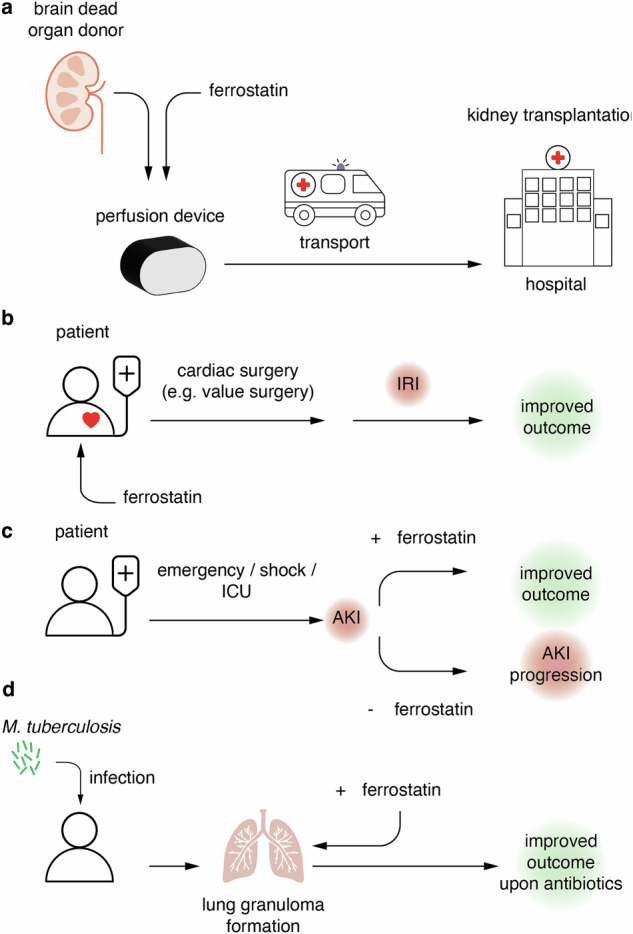
Manifest clinical trials for ferroptosis inhibitors. **a** Ferroptosis inhibition employing a kidney perfusion device upon solid organ transplantation. **b** Ferroptosis inhibitor application before cardiac surgery. **c** Ferroptosis inhibition as a strategy to prevent acute kidney injury (AKI) and nephron loss in intensive care unit patients to improve the AKI-to-chronic kidney disease (CKD) progression. **d** Ferroptosis inhibition as an add on treatment option to anti-tuberculosis antibiotic therapy in critically ill patients may improve respiratory outcomes and limit long-term complications following tuberculosis infection.

As outlined in the previous sections, many clinical conditions may benefit from treatment with ferrostatins. However, clinical trial design may be limited because of the lack of a specific biomarker for ferroptosis in tissues. To consider possible clinical trials despite these circumstances, we recommend to consider the following examples:

The ideal clinically emerging scenario requires a situation in which ferroptosis can be predicted. Solid organ transplantation exhibits one such example (Fig. 4a). This situation offers two possibilities for the application of ferrostatin. First, the brain-dead donor could be treated. Second, the graft, once removed from the donor and perfused in an isolated perfusion device, could be applied with ferrostatins. In both cases, the organ recipient would need to be investigated most carefully for potential side effects. As mentioned above, kidney-transplant recipient patients were already treated with supplementation of Vitamin K, but this was later after transplantation with an entirely different question on progression of calcification [59]. Of course, a stably kidney transplanted patient regularly is on an entirely different and much less aggressive immunosuppressive medication compared to a freshly transplanted individual. And even though the first 3 months following solid organ transplantation may appear arguably different compared with the later life of a successfully transplanted patient, that trial, however, included a kidney transplant cohort without reporting medication-specific side effects [59]. This trial included patients with an estimated glomerular filtration rate (eGFR) as low as 20 ml/min/1.73 m². This also demonstrates that it may be considered safe to treat patients suffering from acute and chronic kidney injury.

Another example of iatrogenic ferroptosis induction is ischemia-reperfusion injury as a consequence of cardiac surgery (Fig. 4b). Upon clinical trial conditions, ferrostatins can be applied before the onset of surgery which precedes the onset of ferroptosis upon reperfusion by as many hours as the surgical procedure may take. As many cardiac patients exhibit compromised renal function, the inclusion of patients with an eGFR or 20 ml/min/1.73 m² in a previous study may also be helpful [59] in the design for this trial, as the common exclusion criterion of an eGFR >30 ml/min/1.73 m² should not apply. This allows to employ the eGFR slope, e.g., assessed over an episode of 6 weeks, alongside with the diagnosis of end stage renal disease (ESRD) and the need for dialysis as secondary and primary endpoints, respectively.

Acute kidney injury is amongst the best studied conditions in ferroptosis research. However, AKI on intensive care units is pathophysiologically different from experimental IRI [121] in its nature as it represents a progressive disease in which nephron by nephron may undergo synchronized regulated necrosis by ferroptosis over a period of several days or weeks [122]. It is an option to treat freshly resuscitated individuals with ferrostatins and enroll them into a clinical trial as soon as they enter the emergency room on an ICU (Fig. 4c). Ferrostatins could be applied in the potentially intubated patients intravenously for a defined period of days (e.g., 5 days) until blood pressure can be sufficiently controlled the situation of cardiovascular shock can be overcome. Primary and secondary endpoints could be defined as composite of death by any cause and requirement for renal replacement therapy (RRT). Alternatively, if no RRT is required, simple serum concentrations of urea and creatinine alongside the estimated glomerular filtration rate (eGFR) could serve as readout systems e.g., 7 or 10 days after discharge from the ICU and 6 months following the enrollment into the trial.

Finally, ferroptosis induction is a pathogenic factor to bacteria [123–125]. One of the most prominent examples causing global disease burden is *mycobacterium tuberculosis* [55]. During granuloma formation and tissue necrosis upon tuberculosis infection of the lungs, ferrostatins might oppose a bacterial virulence factor [125]. In such conditions, ferrostatins would have to be applied for several weeks to potentially unfold their beneficial effects (Fig. 4d). However, upon severe infection, this condition requires weeks of antibiotic treatment in the hospital in almost all cases, since only intravenous antibiotics are available. During such conditions, the enrollment of critically infected patients appears manageable to test the contribution of ferroptotic tissue damage to the overall disease progression.

## Outlook

Advancing therapies to create novel first-in-class drugs for clinical routine requires sequences of mechanistic basic research, biomedical research, translational preclinical research and state-of-the-art drug design. It further requires the definition of clear endpoint studies once the disease patterns are sufficiently understood, and it involves the careful assessment of side effect profiles, especially when novel therapeutic routes are tested in humans. If the clinical readout systems are unequivocally defined, does advancing therapies require a specific biomarker for a sematic term such as “ferroptosis”, or is it sufficient to improve the outcome directly? In our opinion, it is the latter.

For decades, antioxidant treatments have been considered without a clear endpoint to test. “Non-specificity” was a principle of such approaches. Amongst stakeholders in the pharmaceutical industry, this has supported the preconception that radical trapping antioxidants (lipophilic or not), in contrast to kinase inhibitors or monoclonal antibodies, cannot be modern drugs and would be too “unconventional”. A similar preconception was associated with mRNA-based vaccines until the pandemic thought us differently. In our opinion, we should let the ongoing cardiovascular pandemic stimulate our courage to test “unconventional” approaches. Along the same line, the ongoing M. tuberculosis pandemic is another paramount reason to take courage for advanced anti-ferroptosis treatments.
